# Complete genome sequence and phylogenetic analysis of medicinal plant *Abrus cantoniensis* for evolutionary research and germplasm utilization

**DOI:** 10.1002/tpg2.20236

**Published:** 2022-06-24

**Authors:** Shiqiang Xu, Fangping Li, Bingqi Wu, Yu Mei, Jingming Wang, Jihua Wang

**Affiliations:** ^1^ Guangdong Provincial Key Laboratory of Crops Genetics and Improvement, Crop Research Institute Guangdong Academy of Agriculture Sciences Guangzhou 510640 China; ^2^ Guangdong Provincial Engineering & Technology Research Center for Conservation and Utilization of the Genuine Southern Medicinal Resources Guangzhou 510640 China; ^3^ Guangdong Province Key Laboratory of Microbial Signals and Disease Control, Integrative Microbiology Research Centre South China Agricultural Univ. Guangzhou 510642 China

## Abstract

*Abrus cantoniensis* Hance, a native medicinal plant in southern China, is officially recorded in the Chinese Pharmacopoeia. Here, we presented the first high‐quality genome in *Abrus* genus, *A. cantoniensis* genome, as well as the detailed genomic information. The assembled genome size was 381.27 Mb with a scaffold N50 of 18.95 Mb, and 98.97% of the assembled sequences were anchored on 11 pseudochromosomes. The *A. cantoniensis* genome comprised 25,058 protein‐coding genes and 45.12% of the assemblies were repetitive sequences. Comparative genome analysis suggested that chromosome translocation and inversion played an important role in the differentiation of *Abrus*. In addition, 24 toxin‐related genes were identified, which formed two tandem gene clusters on chromosomes 2 and 3. The chromosome‐level genome of *A. cantoniensis* obtained in this work provides a valuable resource for understanding the evolution, active ingredient biosynthesis, and genetic improvement for *A. cantoniensis* and *Abrus* species.

## Core Ideas

- We presented the first high‐quality complete genome in *Abrus* genus, *A. cantoniensis* genome.
- We dissected the toxin‐related genes and detected two tandem gene clusters similar with plant BGCs.
- The research revealed translocation and inversion of chromosomes played an important role in the differentiation of *Abrus*.

**Abbreviations**
BUSCO: Benchmarking Universal Single‐Copy Ortholog
GO: gene ontology
KEGG: Kyoto Encyclopedia of Genes and Genomes
Ks: synonymous substitutions per synonymous site
Mya: million years ago
RNA‐seq: RNA sequencing
tRNA: transfer RNA
WGD: whole‐genome duplication

## 1 INTRODUCTION

*Abrus cantoniensis* Hance, belonging to the genus *Abrus* of the subfamily Papilionideae (Leguminous), is an economically valuable medicinal plant in southern China and officially recorded in the Chinese Pharmacopoeia. The whole plant without pods, named *jigucao* in traditional Chinese medicine, is widely used to treat various diseases such as icterohepatitis, cholecystitis, stomachache, and breast carbuncle (Chen et al., 2009; Yang et al., 2014). As a medicinal and edible plant, *A. cantoniensis* is the main material of herbal tea and commonly used as a folk medicinal diet to treat acute or chronic hepatitis and mastitis. Phytochemical analysis showed that it contains terpenoids, anthraquinones, flavonoids, alkaloids, sterols, and other compounds (Ting‐Shu & Huang, 2019). Within the same genus, *A. mollis* Hance has the similar ingredients and efficacy, which is often used as an alternative material for *A. cantoniensis* (Yang et al., 2015). The seeds of *A. cantoniensis* and *A. Mollis* are poisonous, while rest of the plants are almost nonposionous, so the pods must be completely removed before they can be used as herbal medicine.

In general, the toxicity of *Abrus* species is derived from Abrin protein, a toxic protein composed of two chains (A/B) connected by disulfide bond. Abrin poisoning symptoms usually appear only after the incubation period of a few hours to several days with multiple symptoms such as burning in the mouth, difficulty swallowing, nausea, vomiting, among others (Silva et al., 2005). Genomic studies of the *Abrus* genus firstly focus on *Abrus precatorius* L. Compared with almost nonposionous (except seeds and pods) *A. cantoniensis*, *A. precatorius* is a highly poisonous plant (Hovde et al., 2019). However, the reason for the toxicity differences between these two *Abrus* species remains elusive.

High‐quality genome assembly and annotation were essential references in multiple plant studies (Li et al., 2021). However, a complete and widely recognized reference genome for *A. cantoniensis* was still unavailable. Here, we conducted a series of genomic analyses on *A. cantoniensis* including chromosome‐level assembly, annotations, identification of phylogenetic relationship, gene family analyses, divergence time estimation, and whole‐genome duplication (WGD) analysis. This research will provide important insights as well as resource for future study in *A. cantoniensis*.

## 2 MATERIALS AND METHODS

### 2.1 Plant materials and sequencing

Root, leaf, and stem tissues of ‘JGC‐3’, a popular cultivar of *A. cantoniensis*, were collected from the Guang Zhou City (Tianhe District, 23°9′5.6448′′ N, 113°21′22.8996′′ E) in Guangdong Province, China. High‐quality genomic DNA was extracted using a QIAGEN Genomic Kit. The integrity of the DNA was checked by 0.75% agarose gel electrophoresis. Nanodrop (OD260/280 = 1.8–2.0, OD260/230 = 2.0–2.2) and Qubit were used to detect the DNA purity and the quantity respectively. The genome of *A. cantoniensis* was sequenced using Nanopore GridION X5.

**Core Ideas**

- We presented the first high‐quality complete genome in *Abrus* genus, *A. cantoniensis* genome.
- We dissected the toxin‐related genes and detected two tandem gene clusters similar with plant BGCs.
- The research revealed translocation and inversion of chromosomes played an important role in the differentiation of *Abrus*.

### 2.2 Genome survey and assembly

The genomic size and heterozygosis were estimated by the software GCE (Liu et al., 2013) and Genomescope (Ranallo‐Benavidez et al., 2020). FALCON toolkit v1.8.7 was applied for assembly with the parameters pa_HPCdaligner_option = ‐v, ‐B188, ‐M24, ‐t12, ‐e.75, ‐k17, ‐w8, ‐h1080, ‐T32, ‐l1800, ‐s1000; falcon_sense_option = ‐output_multi, ‐min_idt 0.70, ‐min_cov 2, ‐max_n_read 300, ‐n_core 12; and ovlp_HPCdaligner_option = ‐v, ‐B188, ‐t12, ‐h1080, ‐e.96, ‐k17, ‐T32, ‐l1800, ‐s1000; overlap_filtering_setting = ‐max_diff 60, ‐max_cov 75, ‐min_cov 2, ‐bestn 12, ‐n_core 1(Chin et al., 2016).

### 2.3 Hi‐C experiment and chromosome assembly construction

To further anchor the contigs into chromosomes, fresh young leaves were used to construct a Hi‐C library using a NEBNext Ultra II DNA Library Prep Kit with the enzyme DpnII. Clean data (47.12 GB) was generated for Hi‐C analyses by the Illumina NovaSeq 6000 platform. The placement of contigs was conducted by the pipeline Juicer (Durand et al., 2016) and 3D‐DNA (Dudchenko et al., 2017). Software Juicerbox was implemented for visualization and adjustment (Durand et al., 2016).

### 2.4 Genomic assembly quality assessment

Multiple methods were implemented to assess the accuracy and completeness of the assembled genome. First, the paired‐end reads were mapped to the genome to evaluate its completeness using the BWA‐MEM algorithm with the default parameters (Li, 2013). RNA sequencing (RNA‐seq) data from different tissues were also aligned to the reference genome to obtain the mapping rate using HISAT2 with the default settings (Kim et al., 2015). Second, glycine–cysteing depth scatter plots were used to evaluate any contamination in the sequencing data. Finally, the accuracy and completeness of the genome assembly were evaluated by using the Benchmarking Universal Single‐Copy Orthologs (BUSCOs) to identify the single‐copy genes in the assembled genome with the Embryophyta_odb10 database (Simão et al., 2015).

### 2.5 Gene prediction, repeat element identification

The de novo gene prediction of the genome was performed using AUGUSTUS software (Stanke et al., 2006). The protein sequences from *A. precatorius*, *Arabidopsis thaliana* (L.) Heynh., *soybean* [Glycine max (L.) Merr.], celerabean [*Vigna radiata* (L.) R. Wilczek], grapevine (*Vitis vinifera* L.), and rice (*Oryza sativa* L.) were implemented to homology‐based gene prediction with software GeMoMa.(Jens et al., 2019). Next, the expressed sequence tag and complementary DNA prediction was predicted using PASA. These three annotated evidences were integrated by Software EVM and the output file was used into PASA for three‐round polish and untranslated region prediction (Haas et al., 2008).

The proportion of repeat sequences in plant genomes is not negligible. The regions and types of repetitive sequences of *A. cantoniensis* genomic assembly were identified and soft masked by the software Repeatmasker based on Repbase repetitive sequences database (Jurka, 2000; Chen, 2004).

### 2.6 The annotation of gene function

Functional annotation was achieved by using NCBI BLAST+ v 2.2.2854 (Camacho et al., 2009) with cutoff e‐values of 1 × 10^−5^ and max target sequences of 20 to compare predicted proteins against public databases including SwissProt, Nr (Plant). The best‐hit BLAST results were then considered as the gene functions. Gene ontology (GO) identifiers for each gene were obtained using InterProScan‐5.25‐64.0 by the identification of domains. Several of public databases, including Pfam, SMRT, PANTHER (Zdobnov & Apweiler, 2001), were implemented in this process. The predicted proteins were also submitted to Kyoto Encyclopedia of Genes and Genomes （KEGG） Automatic Annotation Server to get KEGG orthology ID for metabolite pathways  annotation (Kanehisa & Goto, 2000). The noncoding RNA was annotated by comparing against Rfam databases (Griffiths‐Jones et al., 2005). In addition, tRNAscan‐SE and RNAmmer were used to predict transfer RNA (tRNA) and ribosomal RNA of genome (Lowe & Eddy, 1997; Lagesen et al., 2007).

### 2.7 Phylogenetic analysis, estimation of divergence time, and gene family analysis

The gene orthologous as well as single‐copy orthologues genes of 11 species and *A. cantoniensis* were determined by OrthoFinder2 (Emms & Kelly, 2015). MAFFT with option ‐maxiterate 1000 was used to align the coding DNA sequences (CDS) of single‐copy orthologues (Katoh & Standley, 2013). The phylogenetic tree was constructed by the maximum likelihood method using RaxML under the GTRGAMMA model and 100 bootstrap (Stamatakis, 2014). The divergence time of each tree node was inferred using MCMCtree of the PAML (with the following parameters: clock = 2, RootAge = <100.6, model = 7, Bdparas = 110, kappa_gamma = 62, alpha_gamma = 11, rgene_gamma = 23.18, sigma2_gamma = 11.3) (Yang, 2007). The final species tree with *Fragaria vesca* L. as out‐group and the CDS alignments of 626 single‐copy orthologues were used as input of MCMCtree. The phylogeny was calibrated using various fossil records and molecular divergence times were estimated by placing soft bounds at split nodes with the records from the TimeTree database (Kumar et al., 2017). The program CAFÉ with default parameters was used to identify the expanded and contracted gene families for each species (De Bie et al., 2006).

### 2.8 *CYP* gene family analysis

The protein sequences of *A. cantoniensis*, *A. precatorius*, and *Arabidopsis* were used in *CYP* (cytochrome P450; http://dmelson.utmem.edu/biblioD.html) gene family analysis. PF00067 was used for functional domain identification by HMMER (Pfam; v3.3.2; cut‐off at 1 × 10^−90^) (Bateman et al., 2004; Finn et al., 2011). The putative or annotated protein sequences of *CYP* were aligned using MAFFT with default parameters. The *CYP* gene tree was constructed using iqtree with 1,000 bootstrap replicates and JTT+F+R9 model (http://www.iqtree.org/). *Arabidopsis* was designated as the out‐group. Branching nodes with bootstrap values of <80% were treated as collapsed.

### 2.9 Synteny, positive selection genes, and whole‐gene duplication anaylsis

According the above orthologous relationship, Codeml of PAML was used to calculate the selection pressure of the branch of *A. cantoniensis* with branch‐site model (with parameters as follows: Model A, model = 2, NSsites = 2, fix_omega = 0; Model A1, model = 2, NSsites = 2, fix_omega = 1, omega = 1). Subsequently, the significance of positive selection genes was verified by the Chi‐square test (*p* value ≤ .05).

The genomic sequences of *A. cantoniensis, A. precatorius*, and common bean (*Phaseolus vulgaris* L.) were aligned and visualized by MUMMER (Delcher et al., 2003). We performed multiple‐sequence alignment to extract the conserved paralogs of the protein sequences of this data by using OrthoMCL and BLASTp (e‐value ≤ 1 × 10^−5^)(Li et al., 2003). MCScanX was used to identify collinearity blocks (Wang et al., 2012). WGDI was used to identify and extract collinear interval and corresponding gene pairs (Sun et al., 2021). The calculation of Ks (synonymous substitutions per synonymous site) with the method YN00 as well as fourfold synonymous third‐codon transversion (Wang et al., 2014) was implemented to predict the WGD events.

### 2.10 Toxin‐related gene detection

To identify all abrin‐like, prepropulchellin‐like, and agglutinin‐like genes in *A. cantoniensis*, BLAST searches against the assembled *Abrus* genome were performed using published abrin and pulchellin sequences (Hovde et al., 2019). Software iqtree was used in phylogeny and cluster analysis of homologous toxin gene.

### 2.11 Transcriptome data processing and DEGs identification

The alignment of RNA‐seq data was processed by pipeline Hisat2, while the Samtools was used in data sorting and conversion of file formats (Kim et al., 2015). The gene expression statistics was generated by the software FeatureCounts (Liao et al., 2014). The R module DESeq2 was implemented to identify the differentially expressed genes (DEGs)(Love et al., 2014).

## 3 RESULTS

### 3.1 Genomic survey, sequencing, and assembly

**FIGURE 1:**
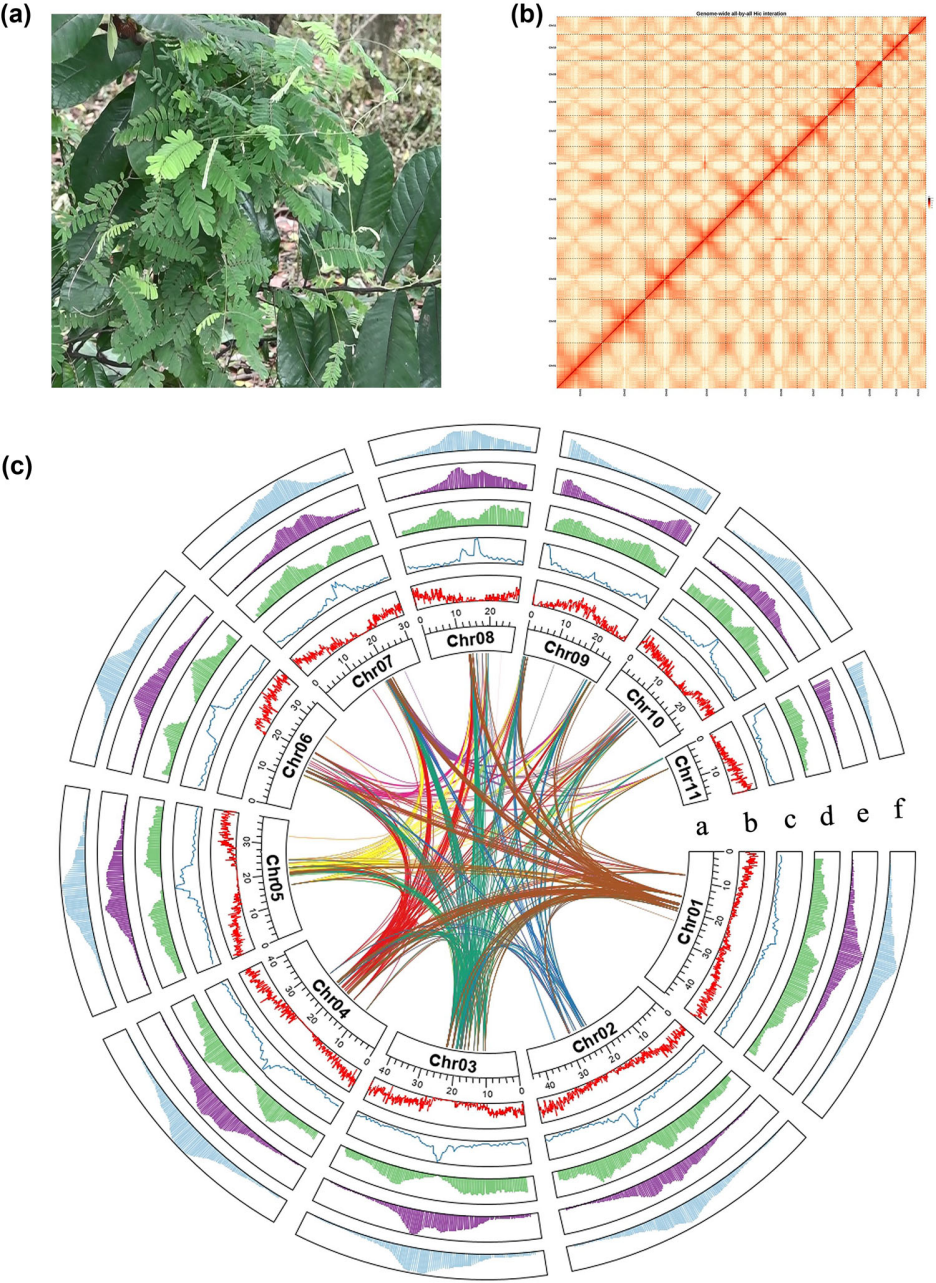
Photo and genomic characteristics of *Abrus cantoniensis*. (a) Photos of *A. cantoniensis*. (b) The Heatmap of HI‐C interaction relationship of *A. cantoniensis*. (c) Characterization of the *A. cantoniensis* genome. The lines inside the circle represented colinear regions within the genome generated mummer comparison. The circle from inside to outside, respectively, represents chromosomes (a), gene density (b), glycine–cysteine content (c), long terminal repeat (LTR)–Copia density (d), LTR–Gypsy density (e), and total LTR density (f)

Based on the total number of *k*‐mers, the genome size and heterozygosity of *A. cantoniensis* were estimated to be 368.8–438.0 Mb and 0.10–0.19%, respectively. (Supplemental Figure S1, Supplemental Table S1). To generate a high‐quality de novo genome assembly of *A. cantoniensis*, 54.53 Gb of long‐read data from Nanopore platform as well as 47.12 Gb HI‐C data were implemented to genome assembly using DNA from the root, leaf, and stem tissues of JGC‐3 (Figure 1a). The final contig level assembly consisted of 46 contigs with N50 length of 18.93 Mb. A total of 98.97% of the assembly was anchored to 11 pseudochromosomes (Figure 1b) whose number of chromosomes was corresponding with the previous karyotype research (Chen, 2009)). The final size of *A. cantoniensis* genome was 381.27 Mb with scaffold N50 18.95 Mb (Table 1). The BUSCO analysis showed that 97.1% of the BUSCO plant genes were identified in the *A. cantoniensis* assembly (Supplemental Table S2).

**TABLE 1:** Genome assembly result

Statistic type	Contig length	Contig no.
	bp	
N50	18,958,252	9
N60	17,463,565	12
N70	17,053,852	14
N80	15,615,987	16
N90	12,547,101	19
Longest	25,624,754	1
Total	381,266,153	46
Length > 1 kb	381,266,153	46
Length > 2 kb	381,266,153	46
Length > 5 kb	381,266,153	46

### 3.2 Repeat sequence identification and genome annotation

The repeat sequences accounted for 45.12% of the assembled genome using a combination of de novo and homology‐based approaches in which 111.85 Mb (29.34%) of the sequences belonged to long‐terminal repeat retrotransposons (Figure 1c; Supplemental Table S3). We also identified 162,743 SSRs in the genome, which contained six kinds of SSR types (Table 2).

**TABLE 2:** Simple sequence repeat (SSR) distribution

Sequence category	Number	Size
		bp
Total examined sequences	29	381,267,853
Total identified SSR	162,743	–
Compound format	16,342	–
Sequences contain SSR	29	–
Sequences contain SSR (>1)	29	–
Unit size 1 bp (repeat number ≥ 10)	94,046	–
Unit size 2 bp (repeat number ≥ 6)	50,626	–
Unit size 3 bp (repeat number > 5)	15,300	–
Unit size 4 bp (repeat number > 5)	1,813	–
Unit size 5 bp (repeat number > 5)	531	–
Unit size 6 bp (repeat number > 5)	427	–

Combining the results of de novo, homolog‐based, and transcriptome‐based predictions, a total of 25,058 protein‐coding genes were annotated across 11 chromosomes of *A. cantoniensis*. The average length and coding sequence size of these transcripts were 3,599.90 and 1,303.16 bp, respectively, with an average of 5.42 exons per transcript (Supplemental Table S4).

Among the protein‐coding genes, 98.32% could be annotated by functional databases (KEGG, SwissProt, GO, NR, and Clusters of Orthologous Groups) (Supplemental Figure S2). The KEGG enrichment analysis showed that 764 genes were involved in carbohydrate metabolism, while only 27 genes were involved in Xenobiotics biodegradation and metabolism (Supplemental Figure S3A,B).

Noncoding RNA was identified based on the alignment Rfam database as well as algorithm from tRNAscan‐SE and RNAmmer, which annotated 273 micro‐RNA, 181 ribosomal RNA, 2,321 small nuclear RNA, and 725 transfer RNA (Supplemental Table S5). Assessment of annotation was implemented through BUSCO. Approximately 94.6% of complete gene elements can be found in the gene set, which indicated that most conservative genes were predicted and reflected the high reliability of prediction result.

### 3.3 Genome evolution and gene family expansion

**FIGURE 2:**
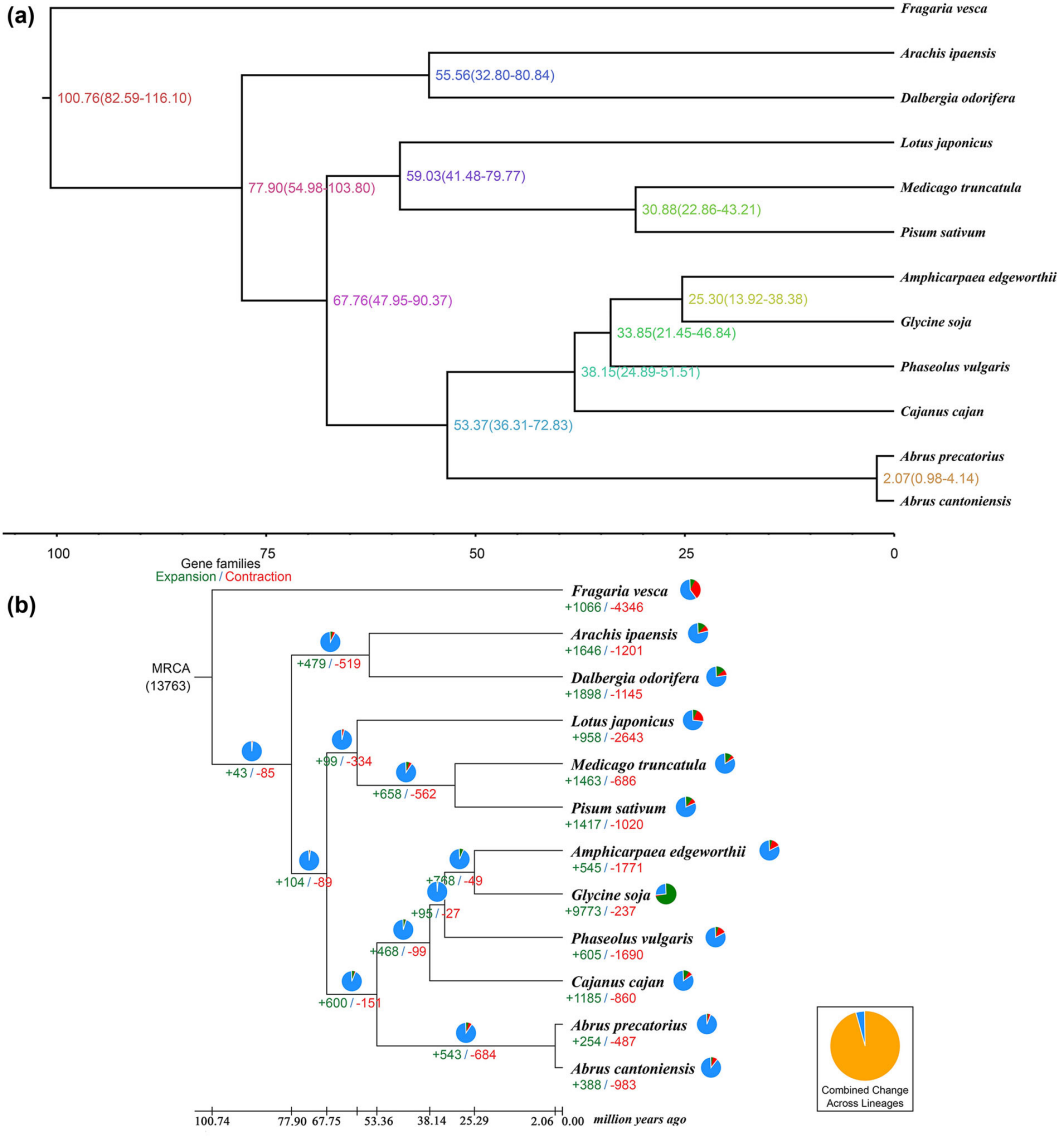
Phylogenomic analysis of genome evolution and gene family expansion. (a) Phylogenomic analysis of *Abrus cantoniensis* and 11 other plant species. The values on the nodes represent the divergence time from now (unit: million years ago, Mya). The divergence time of *A. cantoniensis* and *A. precatorius* was 2.07 Mya. The confidence interval was 0.98–4.14 Mya. (b) Branch length represents differentiation time. The pie chart represents the family data of genes that expand and contract on that branch, with ‘+’ for expansion and ‘−’ for contraction. The expansion and contraction of gene families in various species were illustrated, among which, the 388 gene families of *A. cantoniensis* have expanded, and 983 gene families have contracted during 2.07 Mya

To investigate the phylogenetic relationship between *A. cantoniensis* and other species, Orthofinder2 was used to identify the gene orthologues of 12 species, including *A. cantoniensis*, which generated 626 single‐copy orthologous for the further construction of phylogenetic tree (Supplemental Table S6). This tree showed that the divergence time between *Abrus* and nearest branch of Leguminosae species including *Amphicarpaea edgeworthii* Benth., soybean, common bean, and pigeonpea (*Cajanus cajan* L. Huth) was ∼53.37 Mya (Figure 2a).

Analysis of gene families based on CAFE (De Bie et al., 2006) elucidated that 388 gene families showed significant expansion, while 983 showed contractions in *A. cantoniensis*. The result of Orthofinder2 also elucidated a total of 25,058 genes from *A. cantoniensis* were ortholog genes. Among these genes, 14,724, 189, and 8,883 genes were multiple‐copy orthologs, unique paralogs, and other orthologs, respectively. (Supplemental Table S7).

### 3.4 Synteny, positive selection genes, and whole‐genome duplication anaylsis

**FIGURE 3:**
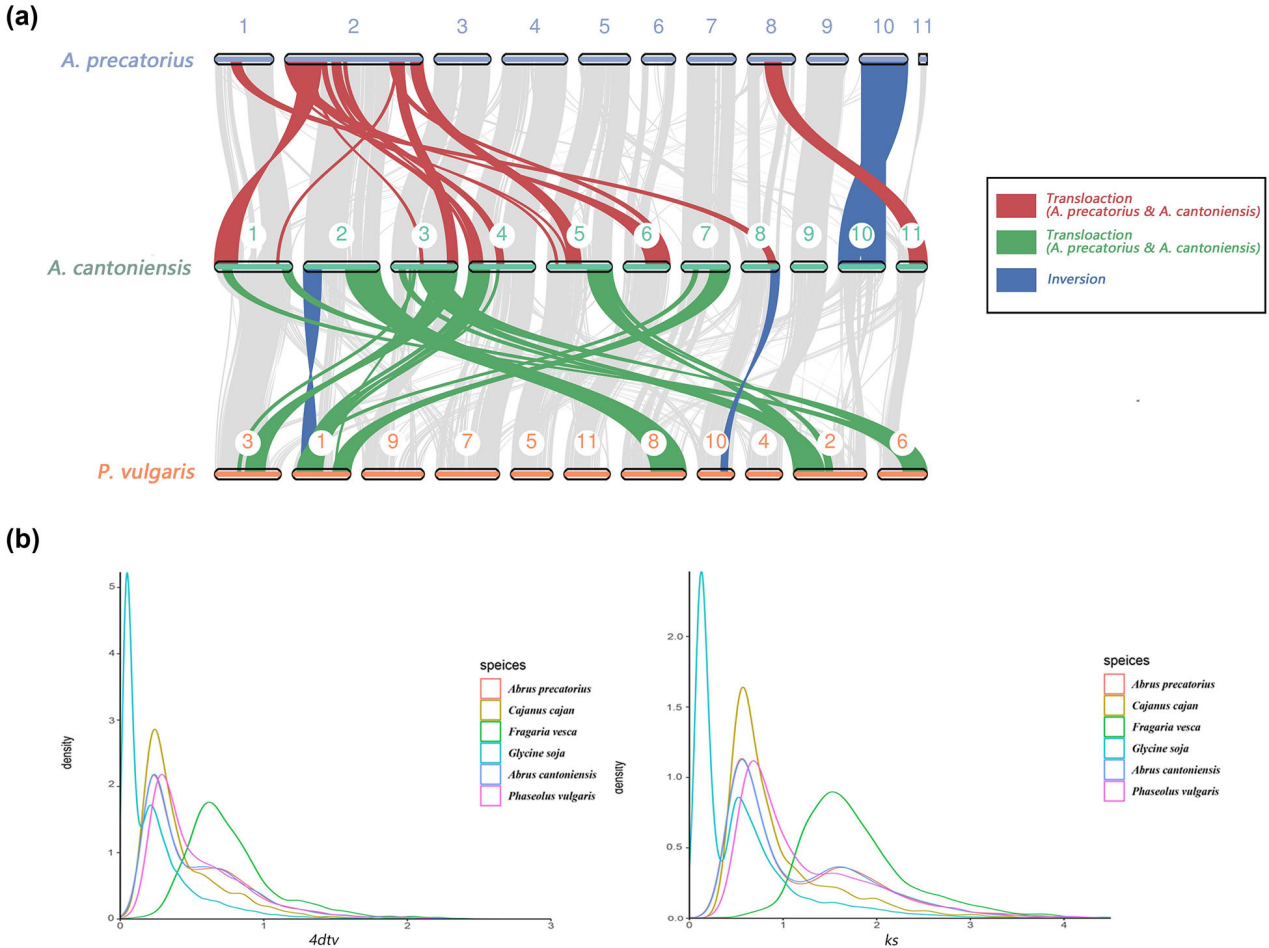
Collinearity and whole‐genome duplication (WGD) events identification of *Abrus cantoniensis* and *A. precatorius*. (a) Gene‐based genomic colinear comparison between *A.cantoniensis*, *A.precatorius* and *P. vulgaris*. (b) fourfold synonymous third‐codon transversion (*4dtv*) and synonymous substitutions per synonymous site (*ks*) plot of WGD events detection of *A.cantoniensis*, *A. precatorius*, and other four plant species

Phylogenetic analysis showed an extremely close evolutionary relationship between *A. cantoniensis* and *A. precatorius*, which was consistent with strong genome‐wide collinearity between these two species. In the meantime, chromosomal translocations were identified in chromosome 10, while multiple large fragment inversion were detected in chromosomes 1, 4, 5, 6, 8 and 11 in the comparison between two species Figure 3a, Supplemental Figure S4A). This phenomenon suggested that the translocation and inversions of chromosomes tended to be a important factor in *Abrus* species differentiation. However, this phenomenon may also be caused by possible assembled errors in the genome of *A. precatorius*.

To further investigate the assembly quality and chromosomal translocations associated with *Abrus*, common bean, an *Abrus* genus closed species with high‐quality genomes (Foucher et al., 2020), was implemented for further genomic comparison analysis. In this analysis, we paired *A. cantoniensis* and common bean chromosomes based on substantial collinearity identified in these two species (Supplemental Table S8, Supplemental Figure S4A). Based on these pairs, multiple segments of collinearity across chromosome pairs were detected (Figure 3a). This suggested that multiple chromosomes in *A. cantoniensis* tended to result from chromosomal structural variations including translocations, recombinations, and ruptures of common bean chromosomes. For example, most of chromosome 11 of *A. cantoniensis* was probably derived from the anterior segment of chromosome 6 from common bean broken in evolutionary events. The posterior segment of chromosome 6 from common bean recombined with the anterior segment of chromosome 2, which participated in the formation of chromosome 3 in *A. cantoniensis* (Figure 3a).

Using the branch–site model (*p* value ≤ .05), we carried out analyses of positive selection in *A. cantoniensis*. The result showed that 52 genes have been positively selected, and 38 genes have been annotated with Swissprot.

To determine the role of WGD events in the differentiation of *A. cantoniensis*, the distributions of Ks as well as four‐fold synonymous third‐codon transversion (4dtv) were estimated based on colinear gene pairs between *A. cantoniensis*, *A. precatorius*, pigeonpea, common bean, soybean, and *Fragaria vesca* (as outgroup). These curves indicated that soybean tended to experience a recent WGD event, which was not detected in *A. precatorius*, pigeonpea, *Fragaria vesca*, and *A. cantoniensis* (Figure 3b).

### 3.5 Analysis of expression characteristics between leaves and stems from *Abrus cantoniensis*

**FIGURE 4:**
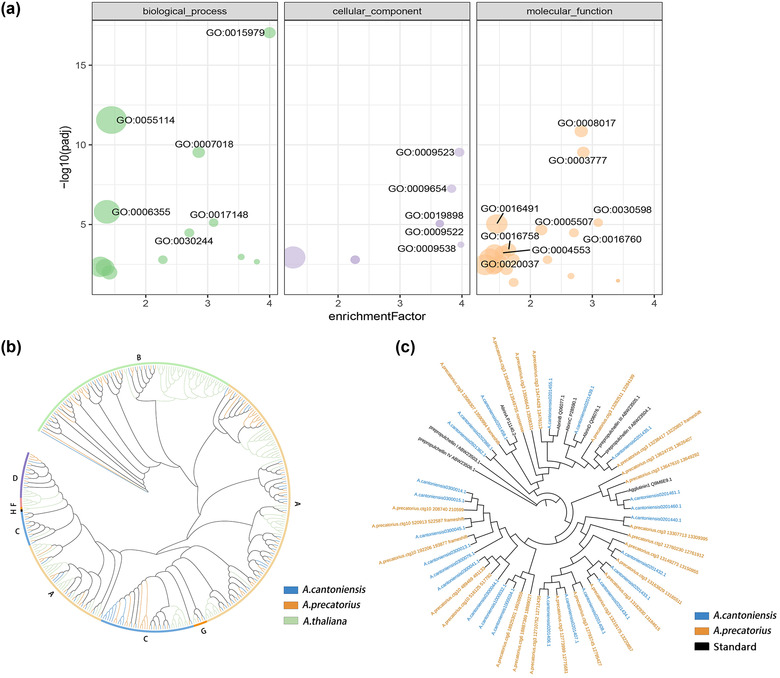
The enrichment analysis of differentially expressed genes (DEGs) between leaves and stems in *Abrus cantoniensis* and the phylogenetic tree about the toxin‐related genes and genes from *CYP* family. (a) The enrichment analysis of the DEGs between leaves and stems in *A. cantoniensis* (GO:0005975: carbohydrate metabolic process; GO:0009623: photosystem II; GO:0016491: oxidoreductase activity). (b) Phylogenetic analysis of *CYP* gene family of *A. cantoniensis*; *CYP* gene family was divided into a, b, c, d, e, f, g subfamilies according to the database. (c) Phylogenetic analysis of toxin‐related genes of *A. precatorius* and *A. cantoniensis*

The expression analysis based on RNA‐seq data indicated that a total of 22,637 genes expressed in the leaves and stems of *A. cantoniensis*. Among these genes, 20,822 were expressed in both types of samples, while 236 and 1,579 genes were respectively uniquely expressed in leaves and stems. Enrichment analysis elucidated that the DEGs from these two plant organs were mainly related to antioxidant and photosynthesis (GO:0005975: carbohydrate metabolic process; GO:0009623: photosystem II; GO:0016491: oxidoreductase activity). These results suggested a stronger antioxidant capacity in leaves than stems in*A. cantoniensis*. By contrast, the difference in the synthesis of secondary metabolites or bioactive substances between leaf and stem was not significant (Figure 4a).

### 3.6 *CYP* gene family analysis

In order to further analyze the evolutionary process of *CYP* gene family of *A. cantoniensis*, phylogenetic trees were constructed for *CYP* gene family members identified in different species. We identified 355 *CYP* sequences from three data sets including 106, 132, and 117 from *A. cantoniensis*, *A.precatorius*, and *A.thaliana*, respectively. According to the category of CYP of Tair database (http://www.p450.kvl.dk/At_cyps/family.shtml; Schuler, 2003), these *CYP* genes can be divided into seven subfamilies (a–f) (Supplemental Table S9; Figure 4b).

### 3.7 Detection of toxin‐related genes

Phylogenetic analysis indicated the correspondence between toxin‐related genes of *A. precatorius* and *A. cantoniensis*. Based on homology search and phylogenetic analysis, a total of 24 toxin‐related genes were identified in *A. cantoniensis* genome, which were detected in chromosomes 2, 3, 5, and 10. Among these genes, 18 clustered in the clades of agglutinin, while six clustered in Abrin and prepropulchlin. Toxin‐related genes (58.3 and 29.16%, respectiviely) belonged to two gene clusters located at chromosomes 2 and 3. The gene cluster on chromosome 2 (12.9–13.7 M) consists of toxic genes from various toxic gene types (Figure 4c; Supplemental Table S10), which suggested that interactions between types of virulence genes had a potential to play an indispensable part in *A. cantoniensis*.

## 4 DISCUSSION

*Abrus* is a genus in Leguminous (butterfly subfamily) with about 12 species of plants and widely distributed in tropical and subtropical regions. *Abrus cantoniensis* is also known as ‘JiGucao’ and widely used as medicine in southern China. Its roots and leaves are used to relieve chronic hepatitis, ascites of liver cirrhosis, stomachache, rheumatism, and arthralgia. The genomic research on *Abrus* genus remained at a preliminary stage.

In this research, we produced the chromosomal‐level genomic assembly of *A. cantoniensis*, the first chromosomal‐level genomic assembly of *Abrus* genus, which will play a vital information resource in *Abrus‐*associated research. This genome will help researchers to further elucidate the molecular mechanism behind the medicinal effects of *A. cantoniensis* and further genetic improvement.

Previous research has released a scaffolded‐level genomic assembly of *A. precatorius*, another species of the genus *Abrus* (Hovde et al., 2019). However, the Hi‐C interaction relationship in this earlier research has not been published. In the meantime, a series of unreasonable chromosomal characteristics in the *A. precatorius* assembly were detected in genomic comparison between*Abrus* species, especially for chromosomes 2 (large segements of chromosomal translocations between multiple chromosomes and extreme long chromosome length) and 12 (extreme short collinearity  and chromosome sequences). Meanwhile, phylogenetics analysis elucidated that the divergence between *A. cantoniensis* and *A. precatorius* occurred at only ∼2 Mya. The reason for such a drastic change in multiple structures of chromosomes in the short independent evolutionary period was worth further research and demonstration. By contrast, the scaffold of chromosomal‐level assembly of *A. cantoniensis* tends to be more well‐proportioned based on an obvious Hi‐C interaction. Additionally, comparative genome analysis also indicated that compared with the previously published *A. precatorius* genome, the complete chromosome‐level *A. cantoniensis* genome had a more reasonable chromosome structure and length scale corresponding to the common bean, a close plant species with high‐quality genomic assembly. These evidences suggested the assembly of *A. cantoniensis* was more suitable to be a better reference genome in research associated with *Abrus*.

The genomic alignment between *A. cantoniensis* and *P. vulgaris* indicated the variation and recombination of chromosomes tended to play an important role in the formation of *Abrus* genus. The similar phenomenon has been observed in evolution of multiple species including banana (*Musa acuminata* Colla), rice (Huang et al., 2020; Martin et al., 2020).

Additionally, we have identified multiple copies of abrin, abrin‐like, and pulchellin‐like genes at the chromosomal level of *A. cantoniensis* in this research. The number and type of these genes were consistent with *A. precatorius*. These toxin‐related genes formed two distinct tandem gene clusters in chromosomes 2 and 3. Recent studies have shown several plant biosynthetic gene clusters (BGCs) of natural products like terpenoids synthesize or phytoalexin formation gene clusters in multiple plants (Miyamoto et al., 2016; Lee et al., 2017). Similarly, in this research, the tandem gene clusters in *A. cantoniensis* suggested that the toxic genes may generally participate in the process of toxin or other natural products synthesis synergistically. The mechanisms behind these results need further investigation.

## 5 CONCLUSION

Here we presented the first high‐quality complete genome in *Abrus* genus, *A. cantoniensis* genome, as well as detailed genomic information. Comparison genomes suggested that the translocations and inversions of chromosomes played an important role in the formation and differentiation of *Abrus* genus. With the help of genes annotated and assembled genome, we dissected the toxin‐related genes in *A. cantoniensis* genome and detected two tandem gene clusters on chromosome 2 and 3. The availability of *A. cantoniensis* complete genome and its genomic information from this work will provide a valuable genome resource for understanding the evolution, active ingredient biosynthesis, and genetic improvement for *A. cantoniensis* and other *Abrus* species.

## AUTHOR CONTRIBUTIONS

Shiqiang Xu: Conceptualization; Investigation; Writing – original draft. Fangping Li: Validation; Visualization; Writing – original draft. Bingqi Wu: Visualization; Writing – original draft. Yu Mei: Investigation. Jingming Wang: Investigation. Jihua Wang: Conceptualization; Investigation; Writing – original draft.

## CONFLICT OF INTEREST

The authors declare no conflict of interest.

## Supporting information

Supplemental Figure S1. The genomic survey analysis result from Genomescope and GCE; A: The genomic survey analysis result from Genomescope B: The 17‐mer depth distribution curve from the module of kmerfreq in GCE.
Supplemental Figure S2. The annotated genes of *A. cantoniensis* among five databases. Venn diagram showed that 24,636 genes were annotated, and 5226 genes were shared among KEGG, GO, KOG, Swissprot and NR.
Supplemental Figure S3. Gene annotation by GO (A) and KEGG (B) database.
Supplemental Figure S4. Synteny of genome sequences between *A. cantoniensis*, *A. precatorius* and *P. vulgaris*; A: Synteny between *A. cantoniensis* and *A. precatorius;* B: Synteny between *A. precatorius* and *P. vulgaris*;
Supplemental Table S1. The result of genomic survey analysis from GCE
Supplemental Table S2. The statistics of BUSCO in the assessment of *A. cantoniensis* genome assembly
Supplemental Table S3. The statistics of the repeats in *A. cantoniensis* genome
Supplemental Table S4. The statistics of gene annotation in *A. cantoniensis* genome
Supplemental Table S5. The statistics of noncoding RNA annotation in A.cantoniensis genome
Supplemental Table S6. The statistics of BUSCO prediction in gene prediction assessment
Supplemental Table S7. The statistics of gene family of twelve species
Supplemental Table S8. Chromosome homology relationship between *A. cantoniensis* and *P. vulgaris* based on collinearity
Supplemental Table S9. The statistics of CYP gene family of three species
Supplemental Table S10 The distribution and classification of toxin related genes in *A. cantoniensis.*

Supporting Information

Supporting Information

## Data Availability

The assembly of *Abrus cantoniensis* generated in this research was available in the NCBI Genome database under BioProject accession number PRJNA818613. The gene annotation and its function annotation file were available in Supplemental File S1 and Supplemental File S2 respectively. The raw sequence data including the reads generated from ONT and illumina platform were accessible with BioSample ID SAMN26867016.
