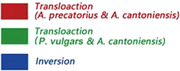# ERRATUM: Complete genome sequence and phylogenetic analysis of medicinal plant Abrus cantoniensis for evolutionary research and germplasm utilization

**DOI:** 10.1002/tpg2.20280

**Published:** 2022-12-01

Shiqiang Xu, Fangping Li, Bingqi Wu, Yu Mei, Jingming Wang, Jihua Wang*

^*^Correspondence

Email: wangjihua@gdaas.cn

The authors noted a few errors in the original manuscript:

In the abstract, the phrase “The assembled genome size was 381.27 Mb with a scaffold N50 of 18.95 Mb” should read “The assembled genome size was 381.27 Mb with a scaffold N50 of 38.03 Mb”

In section 3.1, the phrase “The final contig level assembly consisted of 46 contigs with N50 length of 18.93 Mb” should read “The final contig level assembly consisted of 46 contigs with N50 length of 18.95 Mb (Table 1)”. In addition, the phrase “The final size of *A. cantoniensis* genome was 381.27 Mb with scaffold N50 18.95 Mb (Table 1)” should read “The final size of *A. cantoniensis* genome was 381.27 Mb with scaffold N50 38.03 Mb”

In Figure 3, the legend of FIGURE 3(a) should read