# Supplementary material for: Complete genome sequence and phylogenetic analysis of medicinal plant Abrus cantoniensis for evolutionary research and germplasm utilization
Source: Plant Genome. 2022 Jun 24;15(3):e20236. doi: 10.1002/tpg2.20236 (PMC12807116; doi:10.1002/tpg2.20236)
Supplement: Supplementary file 1 [file TPG2-15-e20236-s003.docx]

**Supplementary Figure 1.** The genomic survey analysis result from Genomescope and GCE; **A:** The genomic survey analysis result from Genomescope **B:** The 17-mer depth distribution curve from the module of kmerfreq in GCE.

**Supplementary Figure 2.** The annotated genes of *A. cantoniensis* among five databases. Venn diagram showed that 24,636 genes were annotated, and 5226 genes were shared among KEGG, GO, KOG, Swissprot and NR.

**Supplementary Figure 3.** Gene annotation by GO (A) and KEGG (B) database.

**Supplementary Figure 4.** Synteny of genome sequences between *A. cantoniensis*, *A. precatorius* and *P. vulgaris*; **A:** Synteny between *A. cantoniensis* and *A. precatorius;* **B:** Synteny between *A. precatorius* and *P. vulgaris*;

**Supplemental Table**

**Supplementary Table 1**. The result of genomic survey analysis from GCE

| **Raw_peak** | **Effective_kmer_species** | **Effective_kmer_individuals** | **Coverage_depth** | **Genome_size** | **a[1/2]** | **a[1]** | **b[1/2]** | **b[1]** | **Heterozygosity** |
| --- | --- | --- | --- | --- | --- | --- | --- | --- | --- |
| 67 | 223623253 | 29653441493 | 67.6519 | 4.38E+08 | 0.0256 | 0.77096 | 0.008971 | 0.410673 | 0.19% |

**Supplemental Table 2.** The statistics of BUSCO in the assessment of *A. cantoniensis* genome assembly

| **Type** | **Number** | **Percent (%)** |
| --- | --- | --- |
| Complete BUSCOs (C) | 1336 | 97.1 |
| Complete and single-copy BUSCOs (S) | 1290 | 93.8 |
| Complete and duplicated BUSCOs (D) | 46 | 3.3 |
| Fragmented BUSCOs (F) | 13 | 0.9 |
| Missing BUSCOs (M) | 26 | 2.0 |
| Total BUSCO groups searched | 1375 | 100 |

**Supplemental Table 3.** The statistics of the repeats in *A. cantoniensis* genome

| **Type** | **Repbase TEs** | | **Mips-REdat TEs** | | **TE proteins** | | **RepeatModeler** | | **Combined TEs** | |
| --- | --- | --- | --- | --- | --- | --- | --- | --- | --- | --- |
|  | **Length (Mb)** | **% in genome** | **Length (Mb)** | **%in genome** | **Length (Mb)** | **% in genome** | **Length (Mb)** | **% in genome** | **Length (Mb)** | **% in genome** |
| DNA | 1450570 | 0.38 | 6221675 | 1.63 | 5209945 | 1.37 | 10402347 | 2.73 | 14168350 | 3.72 |
| LINE | 220510 | 0.06 | 1668015 | 0.44 | 3397410 | 0.89 | 5412609 | 1.42 | 6620719 | 1.74 |
| LTR | 58853479 | 15.44 | 44343613 | 11.63 | 42909766 | 11.25 | 75555271 | 19.82 | 111847033 | 29.34 |
| SINE | 1604 | 0 | 28500 | 0.01 | 0 | 0 | 28361 | 0.01 | 39809 | 0.01 |
| Other | 24398 | 0 | 356441 | 0.1 | 94427 | 0.03 | 0 | 0 | 18303427 | 4.79 |
| Unknown | 0 | 0 | 17196 | 0 | 0 | 0 | 33696295 | 8.84 | 21055071 | 5.52 |
| Total | 60550561 | 15.88 | 52635440 | 13.81 | 51611548 | 13.54 | 123471925 | 32.38 | 172034409 | 45.12 |

**Supplemental Table 4.** The statistics of gene annotation in *A. cantoniensis* genome

| **Specie** | **Gene number** | **ATL(bp)** | **ACL(bp)** | **AEL(bp)** | **AIL(bp)** | **Average exons number per gene** | **Average intron number per gene** |
| --- | --- | --- | --- | --- | --- | --- | --- |
| *Abrus cantoniensis* | 25,058 | 3,599.90 | 1,303.16 | 240.63 | 520.14 | 5.42 | 4.42 |
| *Abrus precatorius* | 24,661 | 3,684.73 | 1,362.76 | 245.2 | 509.47 | 5.56 | 4.56 |
| *Arabidopsis thaliana* | 27,416 | 1,875.54 | 1,220.70 | 237.3 | 158.02 | 5.14 | 4.14 |
| *Glycine max* | 46,535 | 3,630.10 | 1,280.66 | 241.07 | 544.82 | 5.31 | 4.31 |
| *Oryza sativa* | 28,225 | 2,454.91 | 1,086.74 | 254.55 | 418.5 | 4.27 | 3.27 |
| *Vigna radiata* | 26,553 | 3,649.98 | 1,333.65 | 248.42 | 530.23 | 5.37 | 4.37 |
| *Vitis vinifera* | 25,600 | 5,714.31 | 1,341.07 | 256.38 | 1,033.66 | 5.23 | 4.23 |

†ATL: Average transcript length; ACL: Average CDS length; AEL: Average exon length; AIL: Average exon length

**Supplemental Table 5.** The statistics of noncoding RNA annotation in A.cantoniensis genome

| **Type** | **Copy Number** | **Average Length(bp)** | **Total Length(bp)** | **Percentage of Genome(%)** |
| --- | --- | --- | --- | --- |
| rRNA | 181 | 821.36 | 148666 | 0.027868 |
| 18S | 27 | 1864.22 | 50334 | 0.009435 |
| 28S | 23 | 3596.35 | 82716 | 0.015505 |
| 5.8S | 17 | 153.88 | 2616 | 0.00049 |
| 5S | 114 | 114.04 | 13000 | 0.002437 |
| snRNA | 2321 | 107.15 | 248690 | 0.046618 |
| CD-box | 2196 | 105.03 | 230642 | 0.043234 |
| HACA-box | 34 | 129.82 | 4414 | 0.000827 |
| splicing | 91 | 149.82 | 13634 | 0.002556 |
| miRNA | 273 | 122.42 | 33420 | 0.006265 |
| tRNA | 725 | 74.62 | 54102 | 0.010142 |

**Supplemental Table 6.** The statistics of BUSCO prediction in gene prediction assessment

| **TYPE** | **Number** | **Percent (%)** |
| --- | --- | --- |
| Complete BUSCOs (C) | 1300 | 94.6 |
| Complete and single-copy BUSCOs (S) | 1251 | 91 |
| Complete and duplicated BUSCOs (D) | 49 | 3.6 |
| Fragmented BUSCOs (F) | 22 | 1.6 |
| Missing BUSCOs (M) | 53 | 3.8 |
| Total BUSCO groups searched | 1375 | - |

**Supplemental Table 7.** The statistics of gene family of twelve species

| **Species** | **Gene number** | **Gene number in families** | **Unclustered gene number** | **Family number** | **Unique family number** | **Average gene number per family** |
| --- | --- | --- | --- | --- | --- | --- |
| *Abrus cantoniensis* | 25058 | 24422 | 636 | 16010 | 32 | 1.53 |
| *Abrus precatorius* | 24661 | 24568 | 93 | 15979 | 22 | 1.54 |
| *Amphicarpaea edgeworthii* | 27899 | 26069 | 1830 | 16529 | 244 | 1.58 |
| *Arachis ipaensis* | 36862 | 35099 | 1763 | 16207 | 776 | 2.17 |
| *Cajanus cajan* | 28841 | 28148 | 693 | 16338 | 151 | 1.72 |
| *Dalbergia odorifera* | 29854 | 26418 | 3436 | 16538 | 463 | 1.6 |
| *Fragaria vesca* | 23724 | 22595 | 1129 | 14764 | 550 | 1.53 |
| *Glycine soja* | 57957 | 51253 | 6704 | 19063 | 1393 | 2.69 |
| *Lotus japonicus* | 25605 | 24063 | 1542 | 14882 | 322 | 1.62 |
| *Medicago truncatula* | 31128 | 30068 | 1060 | 16145 | 345 | 1.86 |
| *Phaseolus vulgaris* | 25510 | 24639 | 871 | 15939 | 93 | 1.55 |
| *Pisum sativum* | 43756 | 38136 | 5620 | 17709 | 1580 | 2.15 |

**Supplemental Table 8.** Chromosome homology relationship between *A. cantoniensis* and *P. vulgaris* based on collinearity

| **No. of *A. cantoniensis* chromosome** | **No. of *P. vulgaris* chromosome** |
| --- | --- |
| Chr01 | Chr03 |
| Chr02 | Chr01 |
| Chr03 | Chr09 |
| Chr04 | Chr07 |
| Chr05 | Chr05 |
| Chr06 | Chr11 |
| Chr07 | Chr08 |
| Chr08 | Chr10 |
| Chr09 | Chr04 |
| Chr10 | Chr02 |
| Chr11 | Chr06 |

**Supplemental Table 9.** The statistics of CYP gene family of three species

| **SubFamily** | **A** | **B** | **C** | **D** | **F** | **G** | **H** | **Total** |
| --- | --- | --- | --- | --- | --- | --- | --- | --- |
| *Arabidopsis thaliana* | 59 | 36 | 9 | 5 | 4 | 3 | 1 | 117 |
| *Abrus cantoniensis* | 50 | 25 | 22 | 7 | 0 | 2 | 0 | 106 |
| *Abrus precatorius* | 63 | 35 | 23 | 9 | 0 | 2 | 0 | 132 |
| Total | 172 | 96 | 54 | 21 | 4 | 7 | 1 | 355 |

**Supplemental Table 10 The distribution and classification of toxin related genes in** *A. cantoniensis.*

| ID | Chromosome | Start | end | Direction | Toxic types |
| --- | --- | --- | --- | --- | --- |
| A.cantoniensis0201458.1 | Chr02 | 13717172 | 13718860 | + | Abrin |
| A.cantoniensis0202966.1 | Chr02 | 42398379 | 42400196 | + |  |
| A.cantoniensis0501362.1 | Chr05 | 12439371 | 12441158 | + |  |
| A.cantoniensis0201455.1 | Chr02 | 13684165 | 13685850 | + |  |
| A.cantoniensis0201439.1 | Chr02 | 13514381 | 13516069 | - |  |
| A.cantoniensis0201435.1 | Chr02 | 13462072 | 13463762 | - |  |
| A.cantoniensis0201406.1 | Chr02 | 12950214 | 12951896 | - | Agglutinin |
| A.cantoniensis0201407.1 | Chr02 | 13015233 | 13016915 | - |  |
| A.cantoniensis0201408.1 | Chr02 | 13031745 | 13033427 | - |  |
| A.cantoniensis0201432.1 | Chr02 | 13380170 | 13381852 | - |  |
| A.cantoniensis0201433.1 | Chr02 | 13405614 | 13407299 | - |  |
| A.cantoniensis0201434.1 | Chr02 | 13439517 | 13441196 | - |  |
| A.cantoniensis0201440.1 | Chr02 | 13531613 | 13533307 | - |  |
| A.cantoniensis0201460.1 | Chr02 | 13766956 | 13768638 | - |  |
| A.cantoniensis0201461.1 | Chr02 | 13783694 | 13785376 | + |  |
| A.cantoniensis0300013.1 | Chr03 | 228606 | 230282 | - |  |
| A.cantoniensis0300014.1 | Chr03 | 258458 | 260317 | - |  |
| A.cantoniensis0300015.1 | Chr03 | 276124 | 277986 | - |  |
| A.cantoniensis0300041.1 | Chr03 | 535183 | 536850 | + |  |
| A.cantoniensis0300044.1 | Chr03 | 570102 | 571751 | + |  |
| A.cantoniensis0300045.1 | Chr03 | 576789 | 577886 | + |  |
| A.cantoniensis0300076.1 | Chr03 | 832006 | 833668 | - |  |
| A.cantoniensis1000032.1 | Chr10 | 397762 | 399420 | + |  |
| A.cantoniensis1000034.1 | Chr10 | 425404 | 427062 | - |  |
